# Soybeans Grown in the Chernobyl Area Produce Fertile Seeds that Have Increased Heavy Metal Resistance and Modified Carbon Metabolism

**DOI:** 10.1371/journal.pone.0048169

**Published:** 2012-10-26

**Authors:** Katarína Klubicová, Maksym Danchenko, Ludovit Skultety, Valentyna V. Berezhna, Lubica Uvackova, Namik M. Rashydov, Martin Hajduch

**Affiliations:** 1 Department of Reproduction and Developmental Biology, Institute of Plant Genetics and Biotechnology, Slovak Academy of Sciences, Nitra, Slovakia; 2 Institute of Chemistry, Centre of Excellence for White-Green Biotechnology, Slovak Academy of Sciences, Nitra, Slovak Republic; 3 Department of Biophysics and Radiobiology, Institute of Cell Biology and Genetic Engineering, Kyiv, Ukraine; 4 Institute of Virology, Slovak Academy of Sciences, Bratislava, Slovakia; 5 Center for Molecular Medicine, Slovak Academy of Sciences, Bratislava, Slovakia; Lawrence Berkeley National Laboratory, United States of America

## Abstract

Plants grow and reproduce in the radioactive Chernobyl area, however there has been no comprehensive characterization of these activities. Herein we report that life in this radioactive environment has led to alteration of the developing soybean seed proteome in a specific way that resulted in the production of fertile seeds with low levels of oil and β-conglycinin seed storage proteins. Soybean seeds were harvested at four, five, and six weeks after flowering, and at maturity from plants grown in either non-radioactive or radioactive plots in the Chernobyl area. The abundance of 211 proteins was determined. The results confirmed previous data indicating that alterations in the proteome include adaptation to heavy metal stress and mobilization of seed storage proteins. The results also suggest that there have been adjustments to carbon metabolism in the cytoplasm and plastids, increased activity of the tricarboxylic acid cycle, and decreased condensation of malonyl-acyl carrier protein during fatty acid biosynthesis.

## Introduction

Despite the magnitude of the Chernobyl nuclear accident, local flora continues to grow and reproduce in the radio-contaminated soil. Although there has been more than 80 years of research addressing the effects of ionizing radiation on plants [1], the ongoing success of plants in the Chernobyl area was not anticipated. There have been a few molecular analyses of plants grown in the radio-contaminated Chernobyl area, and there is as yet no broad understanding of the mechanisms that underlie survival. Results from analysis of pine trees grown in the Chernobyl exclusion zone indicated genome-wide DNA damage [2], and higher nucleotide diversity in the catalase and glutathione peroxidase genes [3]. There is also DNA-hypermethylation in pine, indicating epigenetic effects in adaptation of pine to a radio-contaminated environment [4], and there appears to be a correlation between genome hypermetylation and radiation sensitivity.

In wheat plants grown in the radio-contaminated Chernobyl area for one generation, the sequences of six microsatellite loci contained complex germline mutations including loci deletion and insertions of unknown origin [5]. In later generations, the wheat DNA showed gains and losses of repeat-DNA and the complete loss of microsatellite bands associated with 13 single-copy monomorphic loci [6]. The progeny of *Arabidopsis thaliana* plants collected from radio-contaminated area around the Chernobyl nuclear power plant were resistant to high concentrations of the mutagens Rose Bengal and methyl methane sulfonate [7]. Additionally, these plants showed significant differences in expression of radical-scavenging and DNA-repair genes upon exposure to mutagens, and a 10-fold lower frequency of extrachromosomal homologous recombination [7]. Analysis of possible molecular mechanisms involved in such resistance revealed a high level of global genome methylation [7].

To complement these genomics and mutagenic studies with proteomics data is the next step in understanding adaptation to permanently increased levels of ionizing radiation. Recently, we analyzed protein abundance in mature seeds harvested from first generation soybean plants grown in radioactive and non-radioactive plots in the Chernobyl area [8]. We found evidence suggesting adaptation to heavy metal stress, protection against radiation damage, and mobilization of seed storage proteins are involved in plant adjustments to increased levels of ionizing radiation [8]. We now extend these data by reporting herein a comparison of changes in protein abundance during seed development of second generation plants grown in either non-radioactive or radioactive plots in the Chernobyl area.

## Experimental Procedures

### Plant Material

Soybean plants (*Glycine max* [L.] Merr.; variety Soniachna) were grown since 2007 in a radioactive field located 5 km from the Chernobyl Nuclear Power Plant (CNPP), near the village Chystogalivka, and in a control field established directly in a non-radioactive area in the town of Chernobyl (Figure S1). The soil in the radioactive field contained 20650±1050 Bq kg^−1^ of ^137^Cs and 5180±550 Bq kg^−1^ of ^90^Sr and in the non-radioactive field 1414±71 Bq kg^−1^ of ^137^Cs and 550±55 Bq kg^−1^ of ^90^Sr. The soil in both fields is sod-podzolic with a loamy-sand texture derived from sandy fluvio-glacial deposits. The soil contains 2.5% organic matter, pH 5.5, and an electric conductivity of 0.20 dS.m^−1^.

During the 2008 growing season, flowers of second generation soybean plants were tagged at opening and developing seeds were collected at four, five, and six weeks after flowering (WAF). These stages were similar to stages undertaken in earlier proteomic studies [9], [10]. Seed width, thickness, length, and fresh and dry weights were measured at each stage. Mature dry seeds were also collected. Protein was quantified from whole-seed homogenates using the Bradford method [11].

### Radioactivity Measurements

Two radioisotopes, γ-ray emitting ^137^Cs and β-ray emitting ^90^Sr, were quantified in mature seeds harvested from non-radioactive and radioactive Chernobyl areas. The ^137^Cs in the samples was quantified using γ-spectrometry as previously described [8]. Measurements employed a semi-conductivity coaxial detector of superpure Germanium, with an energetically resolved 1332.5 keV peak of ^60^Co. The affectivity registration γ-quantum efficiency was 60% (Canberra, Meriden, CT, USA). Instrument calibration used a standard, the gauge EM66 with consistence of 1.1 g cm^3^, and after validation yielded an uncertainty value for ^137^Cs of 7% for 2σ. The gravimetric method for quantifying ^90^Sr using β-radiometry and a low-background UMF-1500M instrument was performed as described previously [12].

The content of ^137^Cs and ^90^Sr radionuclides was measured for shoot system (entire plant without roots and seed pods) and for the seeds. In order to characterize transfer of ^137^Cs and ^90^Sr radionuclides from soil to plant tissues, Transfer Coefficients (TC) were calculated by dividing plant radioactivity by soil radioactivity.

### Total Oil Extraction

Total oil was extracted using a Soxhlet extractor and diethylether as described previously [13] in three independent measurements. One g of seeds was homogenized using mortar/pestle and dried at 105°C for 3 h. Then 250 mL of ether was added and oil was extracted for 16 h. After the extraction, the sample was air-dried until residual ether could not be detected. Oil content was determined gravimetrically.

### Protein Analysis by Two-dimensional Electrophoresis (2-DE)

Proteins were isolated from developing and dry seeds as described earlier [14] with some modifications [9]. Seeds (500 mg) were ground to a fine powder with liquid N_2_ using a mortar and pestle. Proteins were extracted with homogenization media (50% [v/v] phenol, 0.45 M sucrose, 5 mM EDTA, 0.2% [v/v] 2-mercaptoethanol, 50 mM Tris-HCl, pH 8.8), dried under reduced pressure, and resolubilized in 0.5 mL of immobilized pH gradient (IPG) buffer containing 8 M urea, 2 M thiourea, 2% CHAPS, 2% Triton X-100, and 50 mM dithiothreitol (DTT), with gentle agitation for 30 min at 4°C. Insoluble material was removed by centrifugation for 20 min at 14000g at 4°C. Proteins were quantified using the Bradford Reagent (Sigma-Aldrich, Saint Louis, MO) with Bovine Serum Albumin (BSA) as the standard. Protein samples (700 µg) were stored at −80°C until analyzed. The 2-DE was performed as described previously [8]. Shortly, prior to isoelectric focusing (IEF), 2% (v/v) of pH 4-7 carrier ampholytes were added and the samples were loaded onto IPG strips (pH 4 to 7; 17 cm; Bio-Rad, Hercules, CA). The volumes of the samples containing 700 µg of protein were adjusted with IPG buffer to 315 µl. The dehydrated strips were placed onto the sample. After 1 h of passive rehydration, strips were overlayed with mineral oil and placed into the IEF unit (Protean IEF Cell, Bio-Rad, Hercules, CA). After IEF the strips were equilibrated in 2% DTT, and 2.5% iodoacetamide. The strips were placed on top of a 12% acrylamide SDS-gel. Second dimension separations were carried out using Protean II xi Cell (Bio-Rad, Hercules, CA), at 10 mA current for approximately 14 h until dye migrated off of the gel. The samples were analyzed in biological triplicate.

After completion of the 2-DE step, the gels were washed three times for 15 min in deionized H_2_O, and stained overnight in Colloidal Coomassie (20% ethanol, 1.6% phosphoric acid, 8% ammonium sulfate, 0.08% Coomassie Brilliant Blue G-250). The reference (pooled) gel was created by pooling equal amounts of protein (175 mg) from each developmental stage. The 2-DE gels were digitized using a GS-800 Calibrated Densitometer (Bio-Rad, Hercules, CA) at 300 dpi and at 16 bit grayscale (Figure S2).

### Establishment of Protein Abundance Profiles during Soybean Seed Development on Plants Grown in Non-radioactive and Radio-contaminated Chernobyl Areas

Protein 2-DE gels from non-radioactive and radio-contaminated Chernobyl fields were analyzed separately using ImageMaster software 4.9 (GE Healthcare, Uppsala, Sweden) that includes spot detection, quantification, background subtraction, and spot matching. Gels of each developmental stage were matched individually to the reference gel in biological triplicate. A reference gel was obtained by analysis of pooled sample from all investigated stages of seed development. All matched spots were grouped into subclasses. Developmental profiles for a particular 2-DE spot were formed from spot subclasses across all developmental stages.

To ensure high quality of the datasets for comparative analysis, only 2-DE spot that satisfied the following criteria were included: 1) a 2-DE spot must be present in both datasets (i.e. from radioactive and non-radioactive field); 2) in each dataset, a 2-DE spot must be present in at least in two biological replicates and in at least three developmental stages. A total of 211 2-DE spots satisfied these thresholds (Figure S3). Other 2-DE spots were deleted from the dataset. The volumes of all spots that satisfied these criteria were normalized as relative volumes to compensate for small differences in sample loading or gels staining (Table S1). This normalization method divides the volume of each spot by the sum of volumes of all spots in the analysis.

### Protein Identification

All 211 2-DE spots were matched to soybean 2-DE reference maps [9]. This approach provided the identity for 115 2-DE spots (Table S2A). The remaining 96 unmatched spots were subjected to tandem mass spectrometry (MS/MS) based on the MS^E^ method that uses alternate scans at low and high collision energies in order to provide a comprehensive dataset [15]. The MS^E^ was performed as described earlier [16]. Briefly, excised gel plugs were washed, and digested with trypsin (Promega). Following overnight digestion at 37°C, the tryptic peptides were extracted, transferred to a microplate and lyophilized. Tryptic peptides were separated by automated nanoflow reverse-phase chromatography using an Ultra Performance Liquid Chromatography (nanoAcquity UPLC, Waters, Milford, MA, USA) system coupled to a Quadrupole Time-of-Flight (Q-TOF-Premier, Waters, Milford, MA, USA) instrument and analyzed by MS/MS. The resulting data were searched against non-redundant UniProt Glycine max database from March 1 2011(15,416 entries) using Protein Lynx Global Server 2.4 (PLGS). Unidentified MS spectra were searched against non-redundant UniProt Viridiplantae database (March 1 2011; 811,908 entries). Remaining unidentified 2-DE spots were processed against a new release of UniProt Glycine max database from January 24 2012 (46,597 entries). The variable modifications of carbamidomethyl-C, oxidation M, deamidation Q, deamidation N, acetylation N -terminus were specified. One missed cleavage site was allowed. Parameters for accepting the protein identification were; i) detection of at least three fragment ions per peptide, ii) a minimum of two peptides matched to the protein sequence, iii) a 50 ppm tolerance of database-generated theoretical peptide ion masses, iv) PLGS score greater than 15. PLGS score was calculated using a Monte Carlo algorithm in order to analyze all MS data and is a statistical measure of accuracy of assignation. Greater confidence of protein identity implies to a higher score. Using this approach, all remaining 96 2-DE spots were identified (Table S2B).

## Results

### The Soybean Shoot System Accumulated More Radioactivity than Seeds

**Figure 1:**
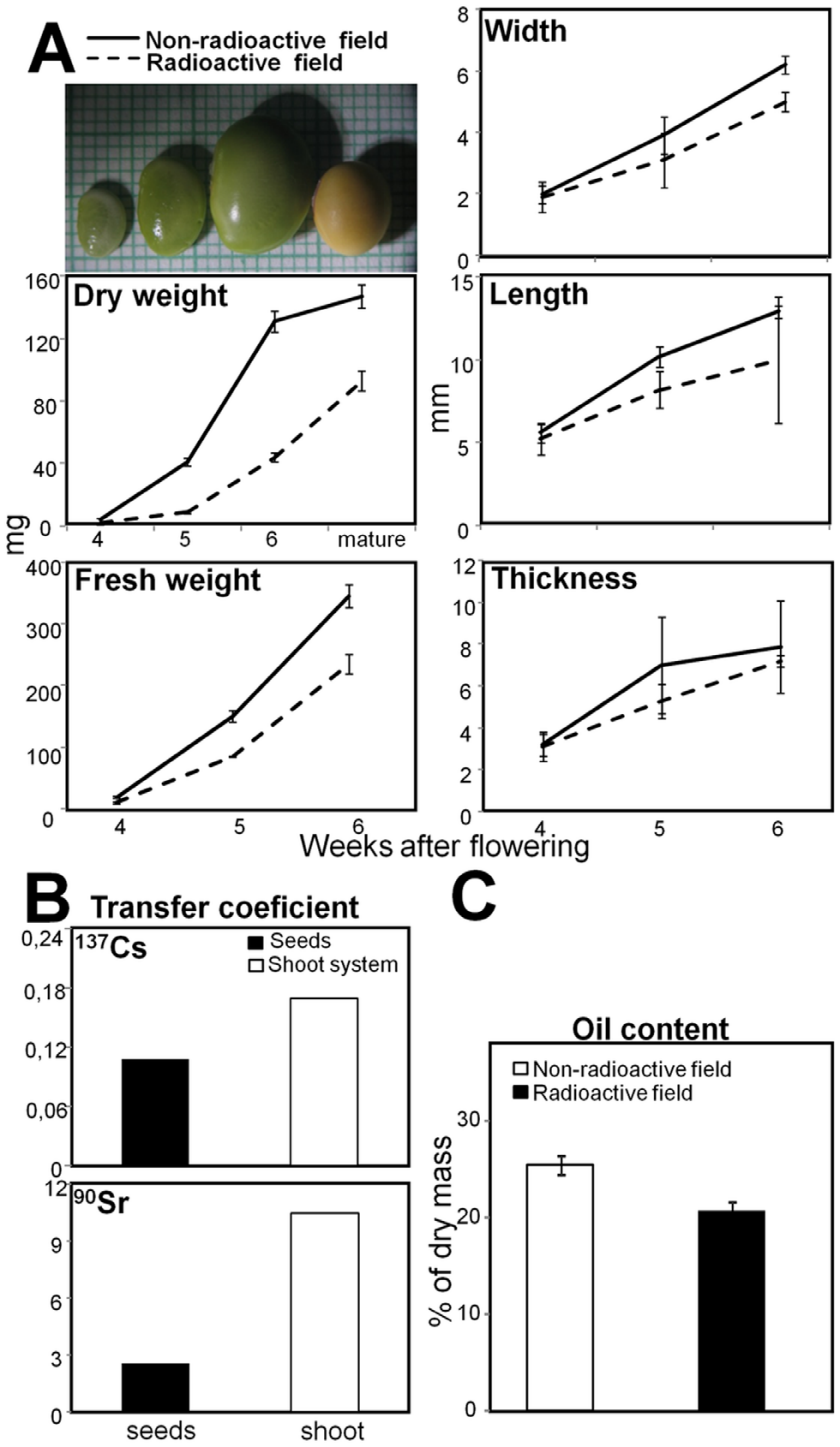
Physiological characterization of developing and dry seeds harvested from non-radioactive and radioactive Chernobyl areas. A. Seeds were characterized for fresh/dry weight, width, length, and thickness. B. Transfer coefficients (TC) calculated for mature seeds and soybean shoot systems (plants without roots and seed pods) harvested from the radioactive area. C. Total oil content in dry seeds. The standard deviations are shown as error dashes.

In the spring of 2008, seeds of the first generation of soybean seeds harvested from the Chernobyl area in 2007 [8] were sown in non-radioactive and radio-contaminated (radioactive) fields (Figure S1). The contents of ^137^Cs and ^90^Sr were 16 and 9 times higher in the radio-contaminated field when compared to the non-radioactive field, respectively (Table 1). The contents of ^137^Cs and ^90^Sr were determined in mature seeds and in the shoot system (whole plants without roots and seed pods) of second generation plants harvested from both fields in 2008 (Table 1). Soil-to-seed/plant Transfer Coefficients (TC) were calculated by dividing the plant tissue radioactivity by soil radioactivity (Fig. 1). It was revealed that shoot systems accumulated more ^137^Cs and ^90^Sr that seeds (Table 1). Additionally, ^90^Sr transferred to the soybean at higher levels when compared to ^137^Cs (Table 1).

**Table 1:** The content of ^137^Cs and ^90^Sr in soil, mature soybeans, and shoot systems (whole plants without roots and seed pods).

		Contents of radionuclide, Bq/kg			
		^137^Cs	^90^Sr	TC for ^137^Cs	TC for ^90^Sr
**Non-radioactive field**					
	Soil	1414±71	550±55		
	Shoot system	27±2	1720±170	0,02	3,13
	Seeds	11±4	90±18	0,01	0,16
**Radioactive field**					
	Soil	20650±1050	5180±550		
	Shoot system	3600±144	54000±2800	0,17	10,42
	Seeds	2130±207	11840±1800	0,10	2,29

The table also shows transfer coeficients (TC) of radionuclides ^137^Cs and ^90^Sr from soil to seeds.

### Soybeans from the Radioactive Chernobyl Area were Smaller, Contained Less Oil, and were Fertile

The flowers from the second generation of soybean plants were tagged after opening and developing seeds were collected precisely at 4, 5, and 6 WAF from non-radioactive and radioactive Chernobyl fields. These stages were selected according to earlier proteomic analyses of greenhouse grown soybeans [9], [10]. Stage 4 WAF corresponds to cell division and 5, 6 WAF correspond to the cell enlargement period [17]. The soybeans width, thickness, length, and fresh weight increased during seed development in the non-radioactive Chernobyl field (Fig. 1), as was described earlier for greenhouse grown soybeans [9]. At cell division stage (4 WAF), the size of the seeds from non-radioactive and radioactive fields was similar. However, during the cell enlargement period (5 and 6 WAF), seeds in radioactive field were significantly smaller (Fig. 1). The dry weight of mature soybeans harvested from the radioactive Chernobyl area was only 63% of those harvested from the non-radioactive area (Fig. 1). Total oil content in mature dry seeds harvested from the radioactive area decreased to 20% from 25% in the seeds harvested from the non-radioactive area (Fig. 1). Dry mature soybeans harvested from the radioactive Chernobyl area were sown the following year (2009) in the same field and produced the third generation of soybean plants (Figure S4).

### The Abundances for 211 Proteins were Characterized during Soybean Seed Development in Non-radioactive and Radioactive Chernobyl Areas

**Figure 2:**
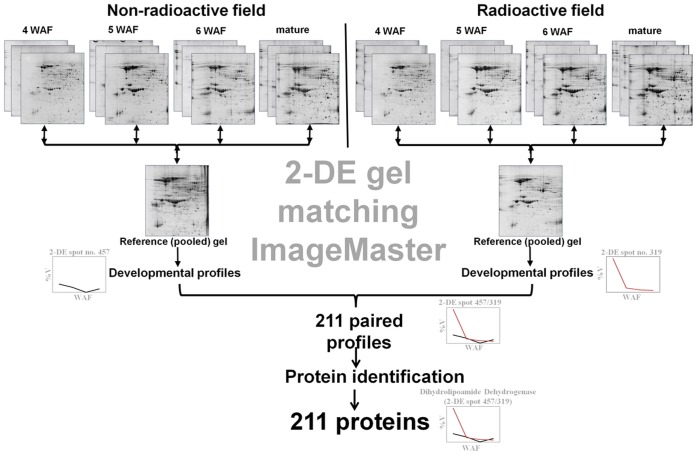
Experimental workflow. Proteins were isolated from developing (4, 5, and 6 weeks after flowering (WAF) and mature soybean seeds harvested from non-radioactive and radioactive Chernobyl areas and analyzed by two-dimensional protein electrophoresis (2-DE) in biological triplicate. Resultant 2-DE gels were matched to the reference (pooled) gels using ImageMaster 4.9 software within each dataset (i.e. seed development in non-radioactive and radioactive fields). This approach resulted in 211 2-DE spots that were matched between seed development in non-radioactive and radioactive Chernobyl areas. These 2-DE spots were identified either by matching to soybean 2-DE reference map (www.oilseedproteomics.missouri.edu) or by tandem mass spectrometry.

The experimental workflow is described in Fig. 2. Developing and mature seeds were harvested from second generation of soybean plants grown in non-radioactive and radio-contaminated experimental Chernobyl fields (Figure S1). Soluble proteins isolated from harvested seeds were resolved using 2-DE (Figure S2), analyzed using ImageMaster software, and quantified 2-DE spots of interest (Figure S3) were subjected to protein identification (Fig. 2). This approach resulted into 211 proteins which abundances were characterized during soybean seed development in non-radioactive and radioactive Chernobyl areas (Fig. 2; Table S2A, S2B; Table S3). The abundance profiles for these 211 proteins are also available at “Plants in Chernobyl” web-based database in user-friendly format http://www.chernobylproteomics.sav.sk/results/5.

**Figure 3:**
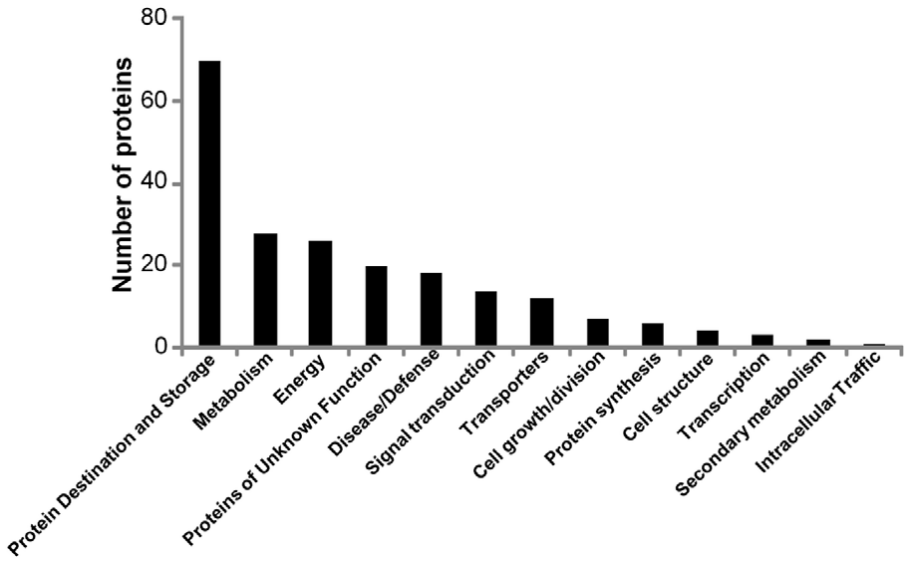
Functional classification of the 211 soybean proteins with paired abundances between soybean seed development in non-radioactive and radioactive Chernobyl fields. The most abundant functional class was proteins associated with Protein destination and storage followed by Metabolic and Energy proteins.

All 211 proteins were sorted into 13 functional categories according to Bevan [18] with some modifications (Fig. 3). The “unclear classification” was merged with “unclassified,” and renamed “proteins of unknown function” (PUF) [19] (Table S2A, S2B). The most populated group of identified proteins were associated with protein destination and storage (70 proteins), followed by 29 proteins associated with metabolism. The third most abundant functional class were proteins associated with energy (25), PUF (21), proteins associated with disease/defense (18) and signal transduction (13). This study also identified 11 Transporters and 7 proteins associated with cell growth/division (Fig. 3).

To determine sub-cellular location of identified proteins, prediction tools TargetP (http://www.cbs.dtu.dk/services/TargetP/), iPSORT (http://ipsort.hgc.jp/), and Pretodar (http://urgi.versailles.inra.fr/predotar/predotar.htmL) were used. From 211 identified proteins 124 were localized in the cytoplasm, 53 in the endoplasmic reticulum/secretory pathway, 20 in the plastids, and 14 in the mitochondria (Table S2A).

### Composite Abundance Profiles Revealed Significantly Decreased Accumulation of β-conglycinins During Seed Development in the Radioactive Chernobyl Area

The composite abundance profiles were established by summing of individual proteins abundances (relative volumes) at each of the metabolic class that contained 20 or more proteins as was shown previously [9], [10], [20]. Only functional classes of Metabolism, Energy, and Protein destination and storage satisfied the criterion (Table S2A). In order to provide specific information about the accumulation of two major soybean SSPs, 15 glycinins and 24 β-conglycinins were extracted from Protein destination and storage functional class and analyzed separately.

**Figure 4:**
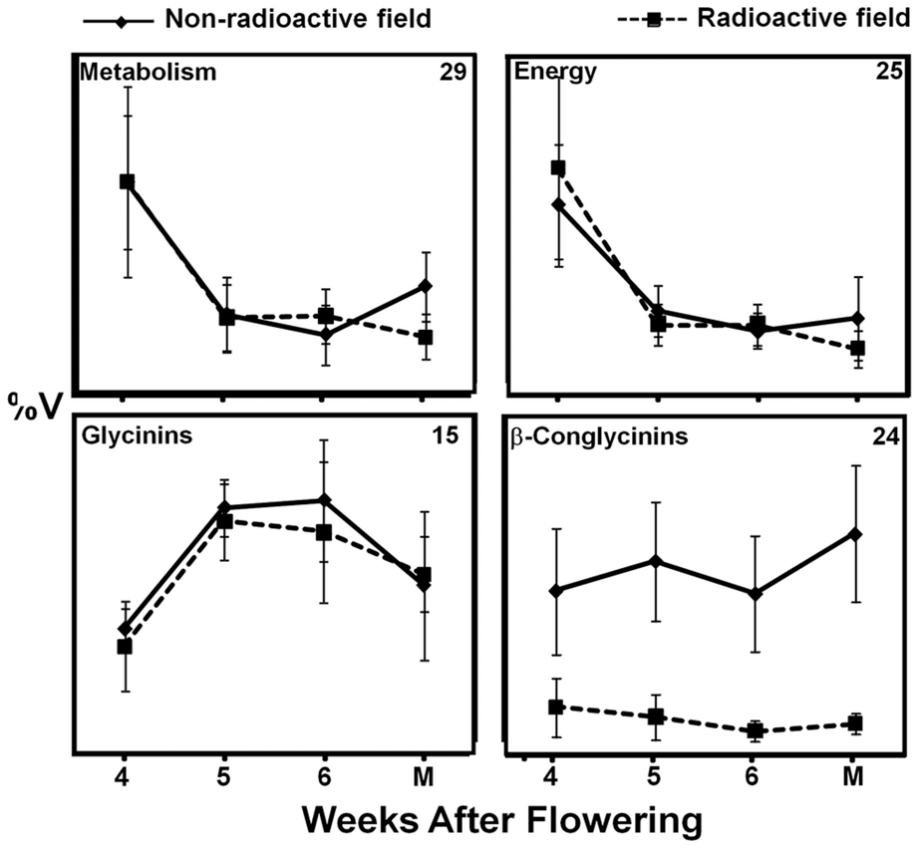
Composite protein abundance profiles for metabolic classes that were established by summing of abundance profiles for individual proteins within metabolic groups Metabolism and Energy and for storage proteins (β-conglycinins and glycinins) during soybean seed development in radioactive (dashed line) and control Chernobyl areas. The number of proteins within each composite protein abundance profile is shown.

Overall abundance of 29 proteins associated with metabolism decreased through soybean seed development from the non-radioactive area (Fig. 4), which is in agreement with previous data obtained for seed development of greenhouse-grown soybeans [9], [10]. The radioactive environment slightly altered the abundance of metabolic proteins at the 6 WAF and mature stages (Fig. 4). However, based on standard deviations, these differences are not significant (Fig. 4). The abundance of 25 proteins associated with energy reactions decreased with seed development from the non-radioactive area (Fig. 4), which is in agreement with previously published data [10]. Growth in the radioactive environment slightly altered the abundance of 25 energy proteins in this study (Fig. 4). However, similar to the metabolic proteins this alteration was not statistically significant (Fig. 4). The abundance of 15 identified glycinin SSPs in non-radioactive field-grown plants increased during seed development, in agreement with previous data [9]. Growth in the radioactive environment changed the abundance profiles of the glycinin SSPs, but the difference was statistically not significant (Fig. 4).

Another type of soybean SSP, the β-conglycinins increase in abundance during seed development in the non-radioactive environment, confirming previous data [9]. Based on standard deviation, the decrease in level of the β-conglycinins is significantly lower in the mature seeds harvested from radio-contaminated plots in the Chernobyl area (Fig. 4).

## Discussion

The aim of this study was to compare the proteome of developing soybean seeds from plants grown in either in radio-contaminated or non-radioactive plots in the Chernobyl area. Seeds harvested from radio-contaminated plots have a specific phenotype; they were smaller and contained less oil. However, to correlate proteomic data with phenotype is difficult due to increased complexity as we climb up “pyramid of life” from genes, through proteins and metabolites, to phenotype [21]. The main obstacle is that changes in protein abundance might not indicate changes in flux of through metabolic pathways [22]. Furthermore protein abundance often does not reflect pathway flux because of kinetic properties [23] or branching and circular nature of metabolic pathways [24]. Several sets of challenges that include technical issues, spatial information about metabolites, and pathway fluxes will need to be solved in order to enable precise correlations of metabolic activities with protein and nucleic acid abundance [25]. Taking into account these limitations, herein we discuss observed phenotypes in relation to the observed proteome changes of soybean seeds harvested from radio-contaminated Chernobyl areas.

### Mobilization of SSP and Adaptation Toward Heavy Metal Stress are Part of Soybean Responses Toward Radio-contaminated Environments for Two Generations

Our previous study showed that mobilization of SSP and altered abundance of proteins associated with defense against heavy metals are part of soybean responses toward radio-contaminated environments [8]. A component of storage reserves, SSPs are important in seed defense against various threats. For instance it was proposed that legume SSPs play a role in seed defense against Bruchids [26] and that 2S albumins are defensive proteins [27]. Furthermore, in the model plant *Arabidopsis thaliana*, the application of salicylic acid resulted in mobilization of SSP during germination [28]. In soybean, the abundance of β-conglycinin SSP was altered by salt stress [29]. The mobilization of SSP was also detected in mature soybean seeds harvested from the first generation of soybean plants grown in the radio-contaminated Chernobyl area [8]. This present study confirms mobilization of SSPs in the seeds harvested from the radio-contaminated Chernobyl area (Table S3) as was shown in our previous study [8]. More specifically, this study detected significantly decreased abundance of β-conglycinins during seed development in radio-contaminated area (Fig. 4). While it is tempting to speculate that decreased levels of the β-conglycinins might be an indirect result of seed size, we do not believe that this is the case. Recently it was shown, that soybean seeds with knockdown synthesis of glycinin and conglycinin developed into similar size and weight as control seeds [30]. However, the reduced size of soybean seeds harvested from the radio-contaminated area agrees with other observations on the effects of ionizing radiation, for instance leaves of *A. thaliana* were smaller because UV radiation reduced epidermal cell expansion [31].

The present study also confirmed our previous data on the involvement of proteins associated with adaptation toward heavy metal stress in soybean responding to the radio-contaminated Chernobyl environment [8]. Cysteine synthase plays an important role in plant adaptation toward heavy metal stress [32]. For instance, transgenic tobacco with overexpressed cysteine synthase tolerated toxic levels of cadmium [33]. In our previous study, we detected a 3-fold higher abundance of cysteine synthase in the seeds of plants grown in radio-contaminated fields [8]. In the present study we also detected an increased abundance of cysteine synthase in developing seeds from plants grown in the radio-contaminated area (Table S3). The dehydrins are also involved in plant protection against heavy metals [34], [35]. Our previous study detected three dehydrins, out of which two exhibited higher abundance in mature soybean seeds harvested from the radio-contaminated Chernobyl area [8]. This present study detected one dehydrin (37495451, 910/617) with higher abundance in developing soybean seeds in the radioactive area (Table S3). These data suggest that increased abundance of heavy metal stress related proteins in developing soybean seeds are part of a soybeans response toward the radio-contaminated Chernobyl environment.

### The Radio-contaminated Environment Altered Carbon Metabolism in Both the Cytoplasm and the Plastids

Several metabolic pathways related to synthesis of nitrogen and carbon storage reserves were detected in green oilseeds previously [36], [9], [10]. In general, oil content in soybeans can be altered by abiotic stresses, for instance by excessive water or heat [37]. Our present experiments show that the radio-contaminated environment significantly reduced oil content in mature soybean seeds from 25% to 20% (Fig. 1). These oil levels are in agreement with previous reports where oil content in soybean seeds was determined to be approximately 20% [38]. To understand molecular mechanisms behind such oil content decreases might help to expand our current knowledge about regulation of oil accumulation in oilseeds that are still not well understood [39].

**Figure 5:**
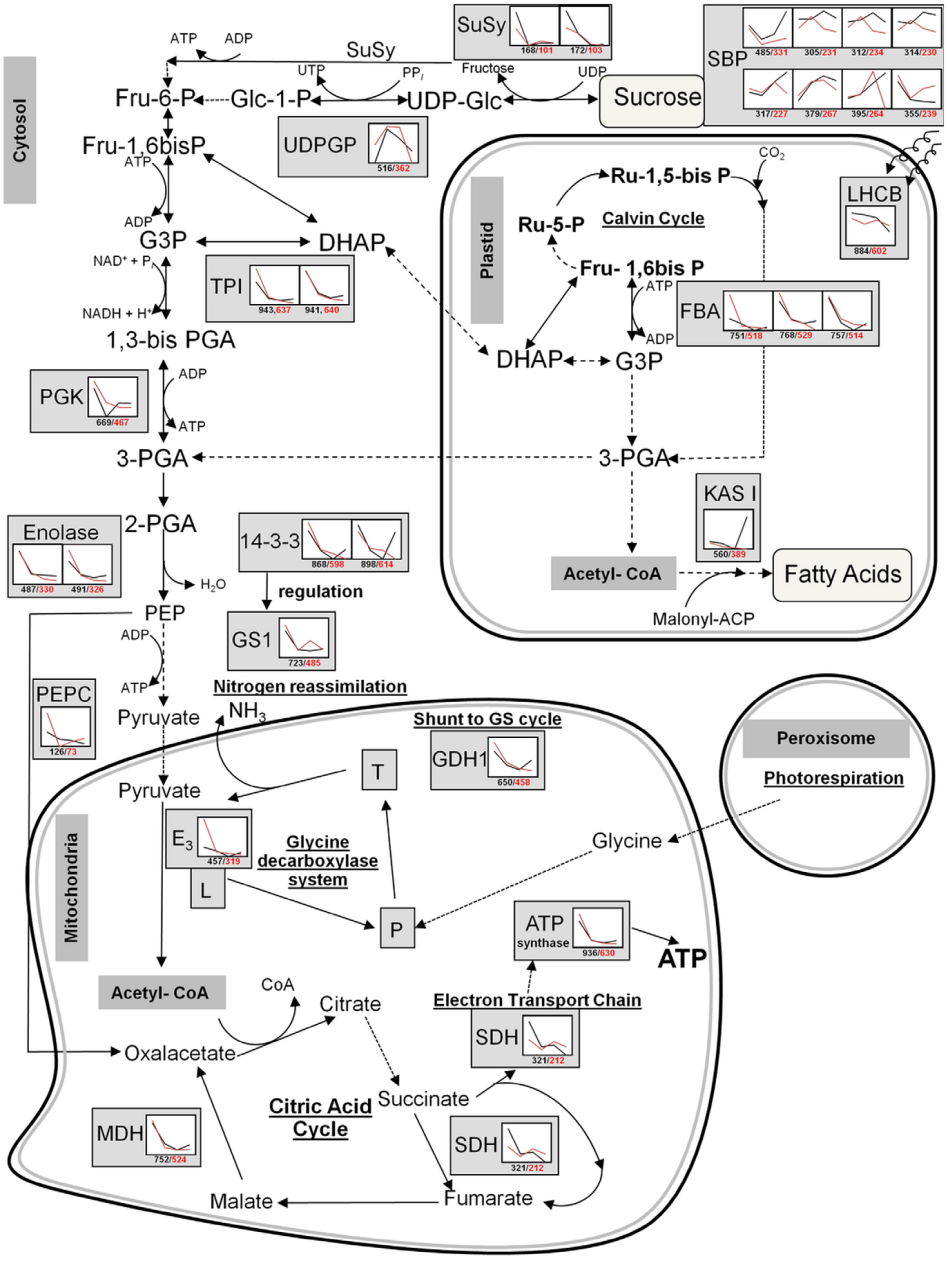
Schematic view of metabolic pathways for carbon assimilation, nitrogen reassimilation, and respiration during soybean seed development in non-radioactive (black) and radioactive (red) Chernobyl areas. Graphs shows abundance of protein spots as relative volumes. Proteins are displayed on corresponding metabolic pathways. Dashed arrows are used when no protein was detected or was detected only during seed development in one area. Abbreviations for metabolites: ACP, acyl carrier protein; ADP, adenosine diphosphate; ATP, adenosine triphosphate; DHAP, dihydroxyacetone phosphate; F-1,6bisP, fructose 1,6 bis phosphate; F-6-P, fructose 6 phosphate; G-1-P, glucose 1 phosphate; G3P, glyceraldehyde 3-phosphate; PEP, phosphoenolpyruvate; 1,3-bis PGA, 1,3 bis phosphoglyceric acid; 2-PGA, 2 phosphoglyceric acid; 3-PGA, 3 phosphoglyceric acid; R-1,5-bis P, ribulose 1,5 bis phosphate; R-5-P, ribulose 5-phosphate; UDP, uridine diphosphate; UTP, uridine triphosphate; UDP-G, UDP-glucose. Abbreviations for enzymes: E_3_ (L), dihydrolipoamide dehydrogenase, L-protein of glycine decarboxylase system; FBA, fructose bisphosphate aldolase; GDH1, glutamate dehydrogenase 1; GS1, glutamine synthetase; KAS1, beta-ketoacyl-ACP synthetase I; LHCB, chlorophyll a/b-binding protein (light-harvesting complex II); MDH, malate dehydrogenase; P, P-protein of glycine decarboxylase system; PEPC, phosphoenolpyruvate carboxylase; PGK, phosphoglycerate kinase; SBP, sucrose binding protein; SuSy, sucrose synthase; SQR, succinate dehydrogenase (ubiquinone); T, T-protein of glycine decarboxylase system; TPI, triose-phosphate isomerase; UDPGP, UTP–glucose-1-phosphate uridylyltransferase.

An important component of carbon incorporation into fatty acids is glycolysis, which was elucidated in the 1940’s [40], [41]. However, relatively little is known about the regulation of glycolysis in plants which is mainly due to the complexity of this pathway in the cytosol and plastids [42], [43]. The initial step – the conversion of sucrose to hexose phosphates occurs in the cytosol. Important components of the sucrose flux system are sucrose binding proteins (SBP) that were firstly identified in soybean cotyledons [44]. The symplastic cell-to-cell sucrose transport occurs through plasmodesmata and apoplastic transport across the plasma membrane [45], [46]. This study identified seven isoelectric species of SBP (GI: 6179947) and one SBP (Uniprot accession no. Q04672) (Table S2). Homology search using Basic Local Alignment Search Tool (BLAST) showed that these accession numbers belong to same sequence of the SBP homolog S-64. The abundances of SBP during soybean seed development in non-radioactive Chernobyl areas follows previously reported abundance profiles during seed development of green-house grown soybean [10]. During seed development in the radio-contaminated area, the abundances of all SBPs, except SBP 355/239, decreased (Fig. 5, Table S3). The abundance of five SBP (395/264, 317/227, 314/230, 305/231, 312/234), decreased from the end of cell enlargement (6WAF) and were detected less in mature seeds (Fig. 5, Table S3). This study identified two isoelectric species (168/101; 172/103) of cytosolic sucrose synthase (SuSy) that catalyzes sucrose (Suc) conversion into uracil-diphosphate (UDP) glucose (Glu) or fructose (Fru) (Table S3). The abundances of both species of SuSy decreased during seed development of soybean in both fields (Fig. 5). However, during the cell division phase of seed development (4WAF), the abundance of both SuSy species (2-DE spots 168/1010 and 172/103), was lower during seed development in radioactive areas (Table S3). The changes in abundances of SBP and SuSy during seed development of soybeans grown in radio-contaminated conditions indicate that sucrose flux into cytosolic glycolysis was altered by the radio-contaminated environment.

The abundances of plastidial light harvesting-chlorophyll a/b-binding protein (LHCB), three isoelectric species of plastidial Fructose-bisphosphate aldolase (FBA) and Beta-ketoacyl-ACP synthetase I (KAS) also suggests changes during carbon assimilation into fatty acids in plastids (Fig. 5, Table S3). Light harvesting-chlorophyll a/b-binding proteins are embedded in the thylakoid membrane in chloroplasts along with chlorophyll form light-harvesting complex II. This study identified spot 884/602 as LHCB that decreased during seed development in both areas and was less abundant during seed development in radioactive areas (Table S3). Fructose-bisphosphate aldolase (FBA) catalyzes the cleavage of Fru 1,6-bis to glyceraldehyde 3-P (GAP) and dihydroxyacetone phosphate (DHAP). Interestingly, this study quantified only plastid forms of FBA (Table S2A). Out of three identified isoelectric species only one (751/518) was differentially abundant between seed development in non-radioactive and radioactive areas (Fig. 5; Table S3). This particular FBA 751/518 was more abundant during seed development in radioactive Chernobyl areas, but only at 4WAF. Beta-ketoacyl-ACP synthetase I (KAS) is involved in the condensation of malonyl-ACP for the growing fatty acid chain [47]. This study identified one isoelectric species of KAS 560/398 but was detected very low in mature seeds harvested from radioactive areas (Fig. 5) as oil in oilseeds accumulates at later stages of seed development [48].

### Tricarboxylic Acid Cycle in Mitochondria was Affected by the Radioactive Environment during Soybean Seed Development

Tricarboxylic acid (TCA) cycle in mitochondria is used to generate energy by the oxidation of acetate into carbon dioxide. Previously it was shown, that the TCA cycle in plants is altered by various stresses. For instance, the TCA cycle was altered by drought stress in maize [49]. The TCA cycle was also altered by salt stress in tobacco [50] and by flooding stress in soybean [28]. Our study detected TCA cycle altered by the radio-contaminated environment (Fig. 5).

The abundance of cytosolic phosphoenolpyruvate carboxylase (PEPC) and mitochondrial dihydrolipoamide dehydrogenase (E_3_) were significantly higher at 4 WAF during seed development in the radioactive Chernobyl area (Fig. 5, Table S3). Protein spot 126/73 was identified as cytosolic PEPC (Table S2). This enzyme was previously detected in developing soybean seeds and it was proposed that PEPC directs a portion of phosphoenolpyruvate (PEP) toward oxaloacetate synthesis in the TCA cycle [51]. The abundance of PEPC (126/73) was high at 4WAF in radioactive areas, then decreased (Table S3).

Similar abundance profiles were detected for 2-DE spot (457/319) which was identified as mitochondrial dihydrolipoamide dehydrogenase (E_3_) of glycine decarboxylase (GI: 9955324) that is also a component of the pyruvate dehydrogenase complex (PDH) [52] (Fig. 5, Table S3). Similarity searches using BLAST revealed that this E3 of glycine decarboxylase (GI: 9955324) is 100% identical to the mitochondrial pyruvate dehydrogenase complex (mPDC) E3 subunit (GI:14916975). Mitochondrial PDC [51] catalyzes the conversion of pyruvate to acetyl-CoA which enters TCA cycle in mitochondria [53]. However, the mitochondrial glycine decarboxylase complex (GCS) can be triggered by high concentrations of glycine molecules cleaved by GCS as they flood out of the peroxisome during photorespiration in C3 plants [54]. High abundance of E3 (GCS) at 4WAF corresponds to increased production of acetyl-CoA but also to increased photorespiration during the cell division phase of soybean seed development in radioactive areas (Fig. 5). Despite that this study failed to quantify mitochondrial citrate synthase (CS), it is tempting to speculate that increased production of oxalacetate by PEPC and acetyl-CoA by GCS resulted in an increased production of citrate via the TCA cycle. The connection between citrate and stress tolerance was proposed previously. For instance, the overexpression of mitochondrial CS improved growth of *Arabidopsis thaliana* in phosphorus limited soils [55]. Citrate was also increased in mitochondria of flooded soybeans [56]. These data indicate that increased production of citrate in mitochondria might be important for successful soybean seed development in radioactive Chernobyl areas.

### Conclusions

The data presented in this manuscript are part of the research on the elucidation of plant adaptation mechanisms toward increased levels of ionizing radiation. The progress of this research can be also followed on the web: http://www.chernobylproteomics.sav.sk where new data are deposited after publication in a user-friendly format. The next step in this research will be to investigate other plant species in radio-contaminated environments and to determine the role of protein post-translational modifications. It will be also necessary to investigate the hypotheses generated by proteomics studies, for instance by the construction of transgenic plants with up/down regulated proteins of interest. All of this effort should lead to the understanding of the molecular basis of plant adaptation toward the environment from increased levels of ionizing radiation. Such information can be than used for the development of strategies of non-food agricultural use of radio-contaminated areas, for instance for biofuel production. With a little of imagination, it is also tempting to speculate that understanding plant adaptation toward ionizing radiation (cosmic radiation) will be necessary for plant cultivation for food purposes during long space missions in the future.

## Supporting Information

Figure S1 **The location of experimental fields in the Chernobyl area.** The non-radioactive field (1414±71 Bq.kg^−1^ of ^137^Cs and 550±55 Bq.kg^−1^ of ^90^Sr) was established directly in remediated area of the town of Chernobyl. The radioactive field was established near the village of Chistogalovka in a radioactive area (20650±1050 Bq.kg^−1^ of ^137^Cs and 5180±550 Bq.kg^−1^ of ^90^Sr), approximately 5 km from the Chernobyl Nuclear Power Plant.
(TIF)

Figure S2 **Protein two-dimensional gels (2-DE) of proteins isolated from soybean developing seeds at 4, 5, and 6 weeks after flowering (WAF) and mature seeds harvested from non-radioactive (A) and radioactive fields (B).** Proteins (500 µg) were separated by isolelectric focusing (IEF) using narrow range IPG strips with pH range from 4 to 7 followed by SDS/PAGE electrophoresis. Resulted 2-DE gels were stained with Colloidal Brilliant Coomassie Blue.
(TIF)

Figure S3 **Reference two-dimensional gel (2-DE) from non-radioactive field with locations of paired protein spots and spot IDs.**
(TIF)

Figure S4 **One-month old soybean plants in radioactive Chernobyl field in 2009.** Harvested mature dry seeds from radioactive field in 2008 were sown in order to test seed fertility.
(TIF)

Table S1 **A.** Relative volumes of 211 protein spots from 2-DE gels of proteins isolated from developing seeds at 4, 5, and 6 weeks after flowering (WAF) and mature seeds harvested from soybean grown in non-radioactive Chernobyl area. The table shows data of biological triplicate analysis. **B.** Relative volumes of 211 protein spots from 2-DE gels of proteins isolated from developing seeds at 4, 5, and 6 weeks after flowering (WAF) and mature seeds harvested from soybean grown in radioactive Chernobyl area. The table shows data of biological triplicate analysis. **C.** Paired 2-DE spots between non-radioactive and radioactive fields.
(XLSX)

Table S2 **A.** The table of 211 proteins identified by either matching to the reference map (115) or by MS^E^ approach (96). The abundance developmental profiles of these proteins were matched between seed development in non-radioactive and radioactive Chernobyl area. The table shows: Protein name; Accession number; NCBI or UniGene Accession Number; Localization, protein subcellular localization; Spot ID non-radioactive: spot number in 2-DE gel of samples from non-radioactive field; Spot ID radioactive, spot number in 2-DE gel of sample from radioactive field; Spot ID reference, spot number in reference soybean 2-DE gel (pI 4-7) available at http://www.oilseedproteomics.missouri.edu/- for 115 proteins identified by comparison to the reference map; PLGS Score; score provided by Protein Lynx software - for 96 proteins identified by MSE; BioWorks Score and MOWSE Score; the scores for spots identified by reference map approach (Hajduch et al., 2005); Pep/Cov, number of identified peptides/coverage in %; Theor MW/pI, theoretical MW/pI value; Exp MW/pI, experimental MW/pI value. **B.** The table of 96 proteins identified by MSE. The table shows: Protein name; Accession number; UniGene Accession Number; Spot ID non-radioactive: spot number in 2-DE gel of samples from non-radioactive field; Spot ID radioactive, spot number in 2-DE gel of sample from radioactive field; PLGS Score; score provided by Protein Lynx Global Server (PLGS); Sequence, sequence of identified peptide; Peptide MH+ (Da), peptide singly protonated mass in Da; z, peptide charge; MH+ Error (Da), peptide singly protonated mass error in Da; MH+Error (ppm), peptide singly protonated mass error in ppm; Modification, modification of identified peptide; Score, PLGS score; Retention Time (min), peptide retention time in min; Intensity, peptide intensity; By Matches, matched "b" and "y" ions to the peptide sequence; Products RMS Mass Error (ppm), peptide root mean square error in ppm; Products RMS RT Error (min), peptide root mean square error of retention time in min.
(XLSX)

Table S3 **The abundances of 211 proteins during soybean seed development in non-radioactive and radioactive Chernobyl areas.** The table shows: Spot ID non-radioactive, spot number in 2-DE gel of samples from non-radioactive field; Spot ID radioactive, spot number in 2-DE gel of sample from radioactive field; protein name; Average, spot relative volume (%V) averages from biological triplicate analysis; STDEV, standard deviation of spot %V; Abundance profiles, protein profiles during seed development in non-radioactive (blue line) and radioactive (red line) Chernobyl areas. The standard deviations are shown as error dashes.
(XLSX)
