# Supplementary figures and images for: Soybeans Grown in the Chernobyl Area Produce Fertile Seeds that Have Increased Heavy Metal Resistance and Modified Carbon Metabolism
Source: PLoS One. 2012 Oct 26;7(10):e48169. doi: 10.1371/journal.pone.0048169 (PMC3482187; doi:10.1371/journal.pone.0048169)

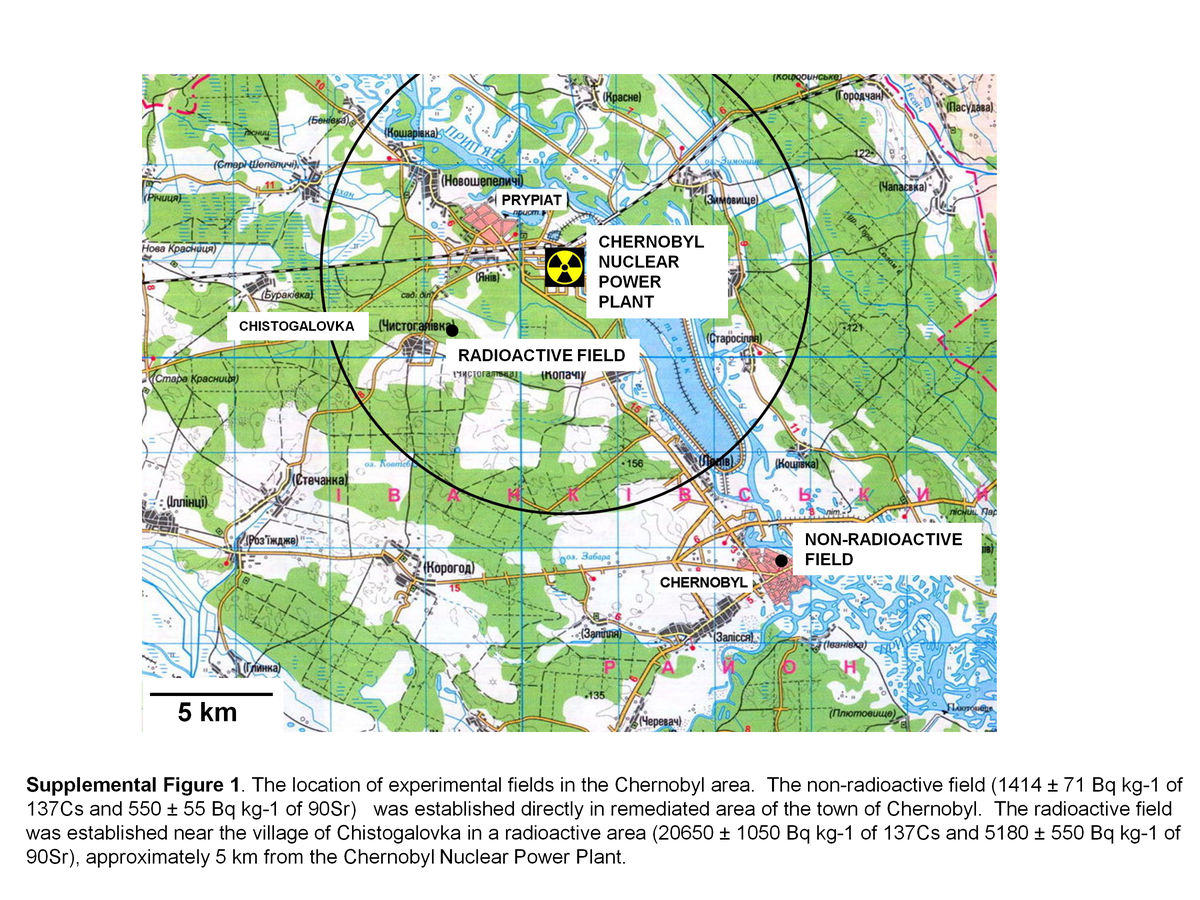

Supplement: Figure S1 — The location of experimental fields in the Chernobyl area. The non-radioactive field (1414±71 Bq.kg−1 of 137Cs and 550±55 Bq.kg−1 of 90Sr) was established directly in remediated area of the town of Chernobyl. The radioactive field was established near the village of Chistogalovka in a radioactive area (20650±1050 Bq.kg−1 of 137Cs and 5180±550 Bq.kg−1 of 90Sr), approximately 5 km from the Chernobyl Nuclear Power Plant. (TIF) [file pone.0048169.s001.tif]

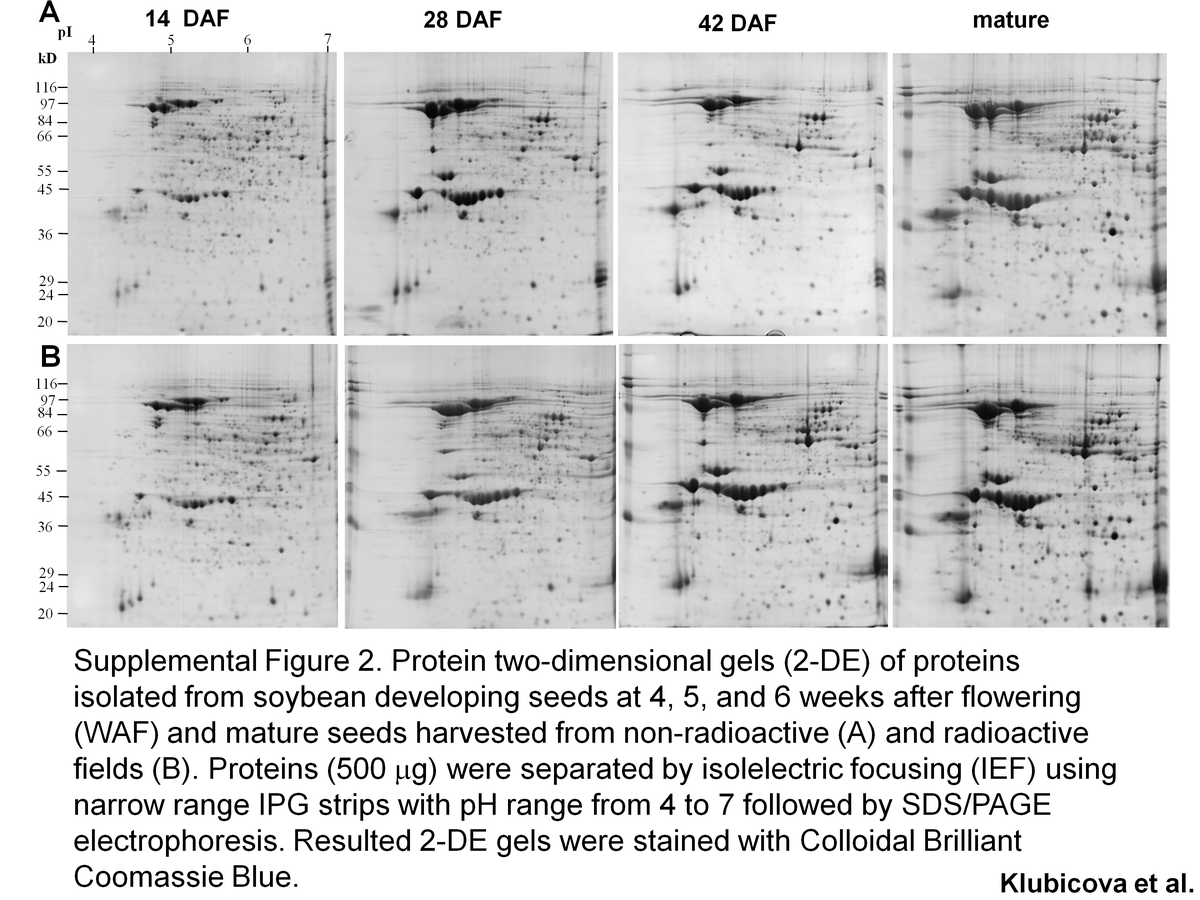

Supplement: Figure S2 — Protein two-dimensional gels (2-DE) of proteins isolated from soybean developing seeds at 4, 5, and 6 weeks after flowering (WAF) and mature seeds harvested from non-radioactive (A) and radioactive fields (B). Proteins (500 µg) were separated by isolelectric focusing (IEF) using narrow range IPG strips with pH range from 4 to 7 followed by SDS/PAGE electrophoresis. Resulted 2-DE gels were stained with Colloidal Brilliant Coomassie Blue. (TIF) [file pone.0048169.s002.tif]

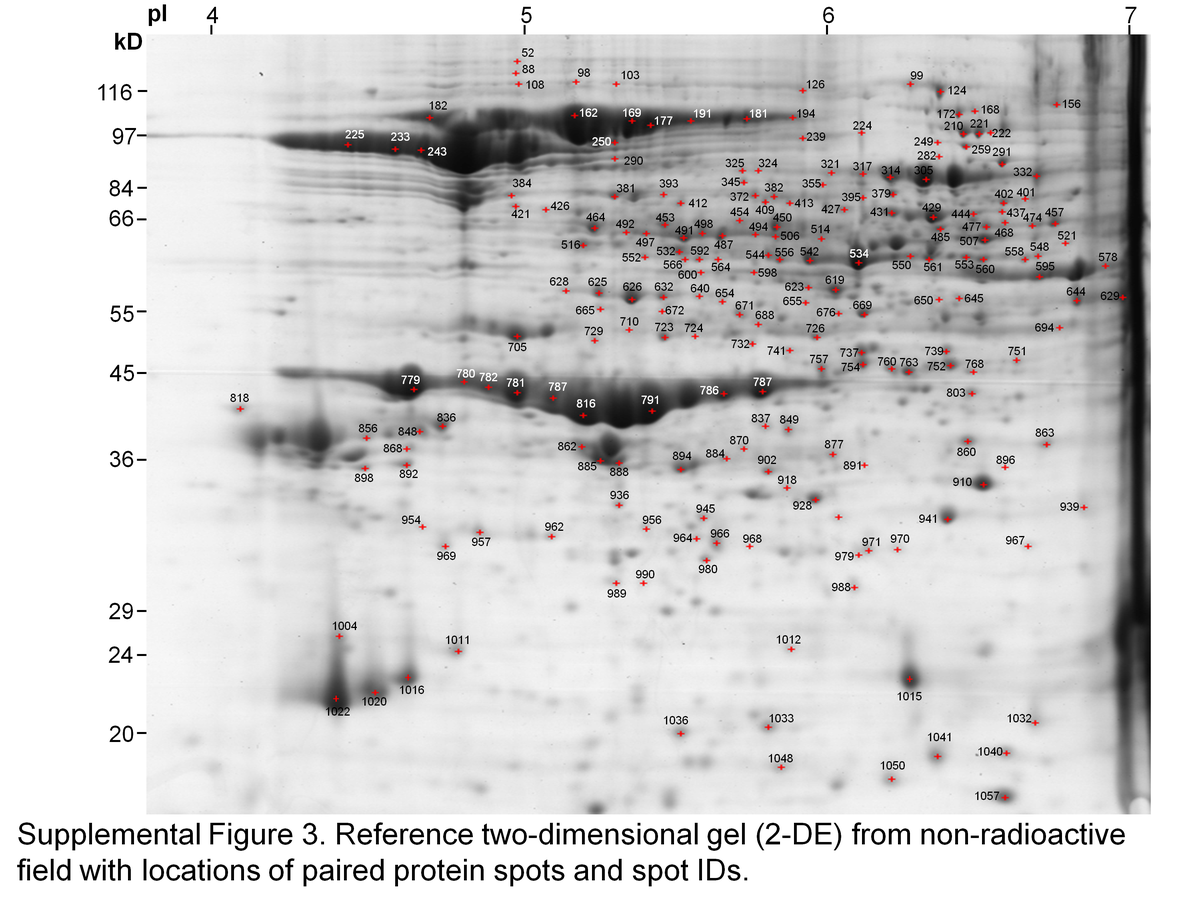

Supplement: Figure S3 — Reference two-dimensional gel (2-DE) from non-radioactive field with locations of paired protein spots and spot IDs. (TIF) [file pone.0048169.s003.tif]

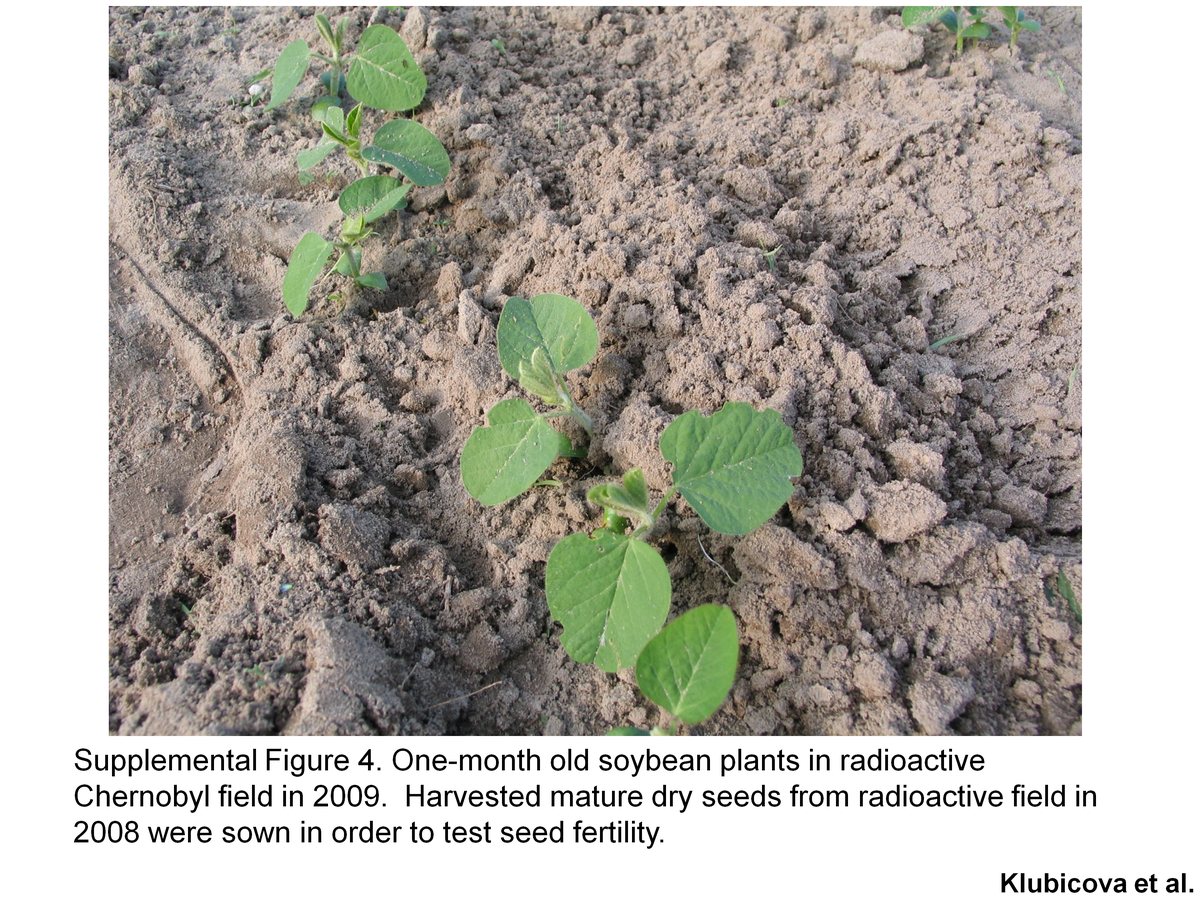

Supplement: Figure S4 — One-month old soybean plants in radioactive Chernobyl field in 2009. Harvested mature dry seeds from radioactive field in 2008 were sown in order to test seed fertility. (TIF) [file pone.0048169.s004.tif]
